# Exercise and Amino Acid Anabolic Cell Signaling and the Regulation of Skeletal Muscle Mass

**DOI:** 10.3390/nu4070740

**Published:** 2012-07-10

**Authors:** Stefan M. Pasiakos

**Affiliations:** Military Nutrition Division, United States Army Research Institute of Environmental Medicine, 15 Kansas Street, Building 42, Natick, MA 01760, USA; Email: stefan.pasiakos@us.army.mil; Tel.: +1-508-233-6474; Fax: +1-508-233-4869.

**Keywords:** mechanotransduction, leucine, hypertrophy, mTORC1, protein synthesis

## Abstract

A series of complex intracellular networks influence the regulation of skeletal muscle protein turnover. In recent years, studies have examined how cellular regulators of muscle protein turnover modulate metabolic mechanisms contributing to the loss, gain, or conservation of skeletal muscle mass. Exercise and amino acids both stimulate anabolic signaling potentially through several intracellular pathways including the mammalian target of rapamycin complex 1 and the mitogen activated protein kinase cell signaling cascades. As novel molecular regulators of muscle integrity continue to be explored, a contemporary analysis of the literature is required to understand the metabolic mechanisms by which contractile forces and amino acids affect cellular process that contribute to long-term adaptations and preservation of muscle mass. This article reviews the literature related to how exercise and amino acid availability affect cellular regulators of skeletal muscle mass, especially highlighting recent investigations that have identified mechanisms by which contractile forces and amino acids modulate muscle health. Furthermore, this review will explore integrated exercise and nutrition strategies that promote the maintenance of muscle health by optimizing exercise, and amino acid-induced cell signaling in aging adults susceptible to muscle loss.

## 1. Introduction

Exercise and amino acids (AA) stimulate skeletal muscle protein turnover by modulating the activity of complex intracellular signaling networks including the mammalian target of rapamycin complex 1 (mTORC1) and the mitogen activated protein kinase (MAPK) signaling cascades [1]. Evidence from *in vitro* and *in vivo* laboratory animal studies demonstrates that mechanical loading initiates muscle protein turnover and anabolic intracellular signaling, thus the mode of exercise performed differentially influences acute and long-term muscle protein responses [2]. Cell membrane stretch-activated calcium channels, intracellular phospholipase D (PLD), and the lipid second messenger phosphatidic acid (PA), have been identified as possible mechanical sensors that may influence muscle intracellular responses to exercise, although the mechanisms by which these unique mechanosensors function in human skeletal muscle have not been determined [3,4]. 

It is also widely accepted that increasing plasma and muscle intracellular AA concentrations stimulate muscle protein synthesis (MPS) [5]. Increasing exogenous AA with exercise potentiates the MPS response initiated by mechanical loading by further enhancing mTORC1 activation through intracellular AA sensing mechanisms including the human vacuolar protein sorting-34 (hVps34) [6,7,8], mitogen activated protein kinase kinase kinase kinase-3 (MAP4K3) [9], the Rag subfamily of Ras small GTPases [10], and also by increasing AA transporter expression [11,12,13]. Although the independent effects of exogenous AA administration and the mechanical stress associated with exercise on intracellular signaling and muscle protein turnover are becoming clear, the cellular mechanisms by which exercise and amino acids combine to contribute to the loss, gain, or conservation of muscle mass remain poorly defined. 

This article provides a concise contemporary review of independent and combined metabolic effects of exercise and AA on intracellular regulators of skeletal muscle mass. Studies identifying novel mechanisms by which contractile forces and AA elicit metabolic responses that may modulate muscle health will be highlighted. Further, this article will explore integrated exercise and nutrition strategies to promote the maintenance of muscle integrity by optimizing exercise and amino acid-mediated cell signaling in aging adults susceptible to muscle loss. 

## 2. Exercise and Intracellular Regulation of Muscle Mass

Skeletal muscle is a highly adaptive tissue, sensitive to mechanical stress. Sustained mechanical loading can elicit muscle hypertrophy, whereas a chronic decrease in mechanical tension can contribute to muscle atrophy [14]. Adaptations in response to mechanical stress are in large part contingent on alterations in MPS, given that a single bout of resistance exercise can result in elevated MPS that persists 48-h into recovery [15]. Recent studies demonstrated that mTORC1 signaling and MPS responses to resistance exercise differs in magnitude and duration when the total volume and load placed on the muscle [16,17,18], length of time that muscle is under tension [19], and the velocity of contractile forces generated (e.g., eccentric and concentric) are manipulated [20]. Although acute anabolic responses to resistance exercise manipulations may vary, meaningful gains of muscle mass are generally observed with most long-term resistance exercise training programs [2]. However, contractile forces generated with steady-state exercise are typically much lower than those observed with resistance exercise so acute and long-term muscle protein responses to endurance-type exercise also likely differ. Nevertheless, studies have demonstrated that prolonged, steady-state exercise does stimulate anabolic intracellular signaling, MPS, and in particular the synthesis of mitochondrial muscle proteins during recovery, a metabolic response that some suggest is essential to promote repair and aerobic adaptations to endurance-type exercise [21,22,23,24]. Regardless of exercise mode, the principal conclusion from these studies is that the mechanical strain associated with the contractile forces generated by exercise is a central physiological driver of protein accretion [14,25]. 

Mechanotransduction is the term used to describe the conversion of mechanical stress into a biochemical signal that triggers anabolic intracellular signaling and MPS [26]. However the mechanisms by which these signals are transmitted are not well described. Baar and Esser [27] first demonstrated that gains in muscle mass were associated with mechanical loading-induced phosphorylation of the 70-kDa ribosomal protein S6 kinase 1 (p70^S6K1^), a signaling protein originally identified as a growth factor (*i.e.*, mitogen) activated protein kinase involved with messenger ribonucleic acid (mRNA) translation. A subsequent report from Bodine *et al*. [28] showed that the activity of p70^S6K1^ and subsequent gains in muscle mass were mTORC1-dependent. Considering that exercise stimulates growth factor secretion, mainly the release of insulin-like growth factor 1 (IGF-1), it was originally hypothesized that autocrine release of IGF-1 in response to exercise transmitted a signal via a membrane bound receptor that activated mTORC1 and subsequently p70^S6K1^ through the insulin/IGF-1 phosphatidylinositol 3-kinase (PI3K) signaling pathway [29]. In theory, muscle hypertrophy would occur when exercise-induced growth factor signaling was stimulated repetitively with habitual exercise training. 

The growth factor-mediated anabolic signaling and hypertrophy paradigm became the accepted model of mechanotransduction regulation of muscle mass. However, using an *in vitro* model, Hornberger *et al*. [26] demonstrated that p70^S6K1^ activation resulting from exercise occurred despite pharmacologic inhibition of IGF-1. For the first time these data suggest that IGF-1 stimulated PI3K signaling may not be necessary to elicit increased mTORC1 activity. Evidence from genetic animal model studies confirmed that mTORC1 and p70^S6K1^ activation and subsequent muscle hypertrophy are, at least in part, independent of IGF-1 stimulated PI3K signaling [30,31]. A recent report from Hamilton *et al*. [32] using intact, non-genetically modified animals further demonstrates the inability of resistance exercise to modulate IFG-1 signaling, as an acute bout of resistance-type exercise failed to activate the IFG-1 membrane bound receptor. Two other recent studies may provide the most compelling evidence to support the IGF-1/PI3K-independent hypothesis by assessing mTORC1 signaling, MPS, and long-term muscle hypertrophy adaptations to varying resistance exercise protocols designed to elicit vast differences in anabolic hormone concentrations [33,34]. In these studies, healthy young men performed two resistance exercise bouts on separate days. One bout consisted of an isolated elbow flexor exercise designed to maintain basal hormone concentrations (low hormone), and the second bout included the same arm exercise on the contralateral arm, immediately followed by heavy leg resistance exercise designed to elicit large increases in anabolic hormones (high hormone). Despite a 10-fold increase in IGF-1 and growth hormone levels in the high hormone state, no acute differences in p70^S6K1^ phosphorylation and MPS were observed between the low and high hormone resistance exercise protocols [34]. Furthermore, overall increases in muscle fiber cross sectional area and muscle strength following 15-weeks of training were not different between high and low hormone resistance exercise protocols [33]. Although the critical role of IGF-1 in the development of maturing tissue cannot be discounted, in the context of mTORC1 and subsequent hypertrophy, these data strongly suggest that mechanisms other than growth factor-induced anabolic intracellular signaling play a major role in mechanotransduction and long-term muscle adaptations to exercise. 

### Intracellular Signaling, Mechanosensing, and the Anabolic Response to Exercise

**Figure 1:**
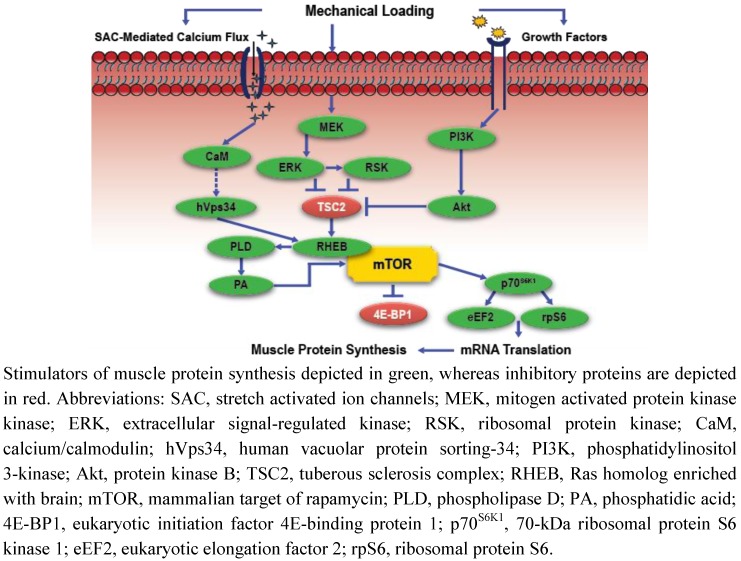
Simplified schematic of mechanotransduction-mediated anabolic signaling.

Intracellular regulation of mRNA translation and MPS responses to exercise are mediated by mTORC1 modulation of the phosphorylation states of two downstream target substrates, p70^S6K1^, and the translational repressor eukaryotic initiation factor 4E-binding protein 1 (4E-BP1) (Figure 1) [1]. However, the MAPK cascade may also play a critical role in skeletal muscle adaptations to exercise, as the activity of this signaling pathway is positively associated with muscle growth, and sensitive to inflammatory, growth factor, and cellular stress responses to exercise [35,36,37,38]. Extracellular signal-regulated kinases 1 and 2 (ERK 1/2), p38 MAPK, c-Jun NH_2_-terminal kinases (JNK), and ERK 5 are the four primary components of the MAPK signaling cascade [35]. Drummond *et al*. [39] was the first to demonstrate in human skeletal muscle that maximal anabolic responses to resistance exercise are, in part, dependent on the co-activation of the MAPK and mTORC1 signaling cascades. Moore *et al*. [40] confirmed these findings in human muscle, as enhanced phosphorylation of ERK 1/2 and the 90-kDa ribosomal S6 kinase (p90^RSK^) were demonstrated with concomitant elevations in MPS and mTORC1 activation during recovery from high-volume, single-joint resistance exercise. Elevated p38, JNK, and ERK 1/2 phosphorylation were also recently demonstrated in human skeletal muscle during the first several hours of recovery from high-power, multi-joint resistance exercise [41]. The authors of this study hypothesized that MAPK signaling responses during the early stages of recovery from high-intensity resistance exercise may contribute to long-term muscle adaptations to resistance training. In support of this hypothesis, the relationship between MAPK and mTORC1 signaling was recently demonstrated using a less traditional non-physiological protocol to induce muscle growth, further suggesting that MAPK signaling is intricately involved in muscle hypertrophic responses to exercise [42]. Miyazaki *et al*. [42] used a 10-day synergist ablation mechanical overload model to induce hypertrophy in laboratory animals. Muscle hypertrophy, increased MPS, and PI3K-independent elevations in p70^S6K1 ^phosphorylation (*i.e.*, increased p70^S6K1^ phosphorylation occurred before elevations in PI3K signaling) were observed in as little as 1-day of mechanical stress, with concomitant increases in the phosphorylation of mitogen activated protein kinase kinase (MEK 1/2), ERK 1/2, and the tuberous sclerosis complex 2 (TSC2), a key substrate for MEK/ERK signaling. Considering the TSC2 complex is an upstream regulator of mTORC1 and MPS, these data support the hypothesis that mTORC1 anabolic signaling responses to exercise are co-regulated by the MAPK pathway. 

Based on those findings, it appears that initial MAPK, mTORC1, and MPS responses to exercise are IGF-1/PI3K-independent. However, the precise mechanism by which mechanical stressors initiate muscle anabolism is still undetermined. Spangenburg and McBride [43] demonstrated that pharmacologic inhibition of stretch activated ion channels (SAC) in laboratory animals blocked resistance exercise-induced p70^S6K1^ phosphorylation [43]. Intracellular calcium flux is regulated in part by the SAC, and elevations in intracellular calcium may stimulate a calcium/calmodulin interaction with hVps34 (CaM complex/Vps34), which subsequently activates mTORC1 (3-h post-exercise) in the presence of contractile-induced elevations in intracellular AA levels [8]. Therefore, SAC-mediated increases in intracellular calcium levels may function as a critical mechanosensing mechanism contributing to the intracellular anabolic response to exercise. In fact, increasing intracellular calcium using a calcium ionophore in isolated muscle cells has been demonstrated to independently stimulate MPS [4]. Mechanically stretching these muscle cells with concomitant increases in intracellular calcium levels produced a more pronounced stimulation of MPS [4]. However, mechanical activation of mTORC1 signaling was also demonstrated despite when chelation induced a dramatic reduction in intracellular calcium concentrations [26]. Bodine *et al.* [28] also demonstrated that blockade of CaM using pharmacologic calceurin inhibitors, cyclosporin A and FK506, failed to inhibit muscle hypertrophy. However, the concept of calcium-mediated regulation of skeletal muscle mass remains contested [44]. Nevertheless, these findings suggest the existence of other potential mechanosensors, besides SAC-regulated calcium flux, which also act to trigger the anabolic signaling response to exercise. 

The phospholipid enzyme PLD and subsequent product PA may also play a role in mechanotransduced activation of mTORC1 signaling. In a series of *in vitro* and *in vivo* laboratory animal experiments, Hornberger *et al*. [3] demonstrated that: (1) elevations in PA concentrations activated mTORC1 signaling; (2) the PLD isozymes (PLD1 and PLD2) are localized at the z-line of the sarcomere, a central site for contractile force transmission; (3) mechanical stress via eccentric loading stimulated PLD activation, PA accumulation, and mTORC1 signaling; and (4) pharmacologic inhibition of PLD blocked mechanical stress-induced elevations in PA concentrations and mTORC1 signaling. In a subsequent study, O’Neil *et al*. [45] demonstrated that mechanical stress-induced PA accumulation was necessary to stimulate mTORC1 activity and consequent muscle hypertrophy using a similar scientific approach. Furthermore, anabolic signaling responses to elevations in PA concentrations were independent of IFG-1/PI3K signaling, as growth factor signaling was blocked pharmacologically. The mechanism by which this occurs appears to be through a direct activation of the mTOR kinase, as PA competitively binds with the FKBP12-rapamycin binding domain on mTOR [46]. These data indicate that mTORC1 activation in response to mechanical loading is PLD-dependent, which may, in part, contribute to exercise-induced muscle hypertrophy. Although these mechanisms will likely be explored in greater detail in future studies, it is important to note that PA-mediated anabolic intracellular signaling has not been thoroughly examined in human skeletal muscle. Furthermore, though studies suggest that PLD and subsequent PA serve a critical regulatory role in mTORC1 signaling following resistance exercise, it must be recognized that stimulation of mTORC1 in response to other mitogen stimuli is also PLD/PA-dependent [47]. 

## 3. Amino Acid Regulation of Muscle Protein Synthesis

Skeletal muscle protein turnover is also regulated by circulating AA concentrations [5]. Increasing plasma and muscle intracellular AA above fasting levels promotes a robust anabolic response that generally exceeds those elicited by exercise performed in the absence of nutrition [1]. The anabolic response is transient, with MPS reaching maximal stimulation within 2 h after consuming AA before rapidly returning to postabsorptive levels under resting conditions [48,49]. The stimulation of MPS in response to AA are dependent on the availability of essential amino acids (EAA), as non-essential amino acids are not required to stimulate MPS [50]. Although increasing levels of each EAA is required to maximize human MPS [5,51], the branched-chain amino acid (BCAA) leucine is particularly important, as several *in vitro* and *in vivo* animal studies have demonstrated that leucine independently stimulates MPS [52,53,54].

The quantity and quality of AA provided, and the conditions during which AA are administered, dictate the skeletal muscle anabolic response to AA. Reports indicate that MPS at rest and in recovery from resistance-type exercise reaches maximal stimulation after consuming 10 g of EAA with an amino acid profile consistent with a 20 g serving of high-quality protein [55,56]. Increasing the concentration of leucine within a maximal dose of EAA does not confer a greater anabolic stimulus at rest and following resistance-type exercise [57,58]. Under those conditions, intracellular AA transport appears saturated, as consuming a leucine-enriched maximal dose of EAA (3.5 g of leucine within 10 g of EAA) stimulates leucine transport to the muscle but does not enhance muscle intracellular leucine concentrations [57,59]. However, consuming a maximal dose of EAA that is enriched with leucine under conditions of increased metabolic demand such as during steady-state exercise may spare endogenous protein and enhance MPS in recovery [60]. Furthermore, consuming a sub-optimal dose of protein that is enriched with leucine following a bout of resistance exercise also appears to confer a similar anabolic stimulus when compared to a maximal dose of high-quality protein [13]. Leucine-enriched AA nutrition may also benefit older adults and contribute to the conservation of skeletal muscle mass by overcoming the anabolic resistance to consuming protein that is typically observed in aging skeletal muscle [61,62]. 

EAA and particularly leucine act as nutrient signals stimulating MPS by triggering critical steps involved with mRNA translation through the mTORC1 signaling network [63,64]. Transient elevations in plasma insulin concentrations secondary to AA administration [52,65], with concomitant changes in the expression and activity hVps34 [6,7], MAP4K3 [9], and subsequent long-term adaptations of the Rag GTPases [10,66], play a central role in the regulation of AA-induced mTORC1 signaling and MPS. Recent studies also suggest that the function of specific AA transporters contribute to the regulation of MPS in response to various stressors by modulating AA flux, ultimately enhancing muscle intracellular AA availability [11,12,67]. 

### Amino Acid Sensing, Transport, and Anabolic Intracellular Signaling

**Figure 2:**
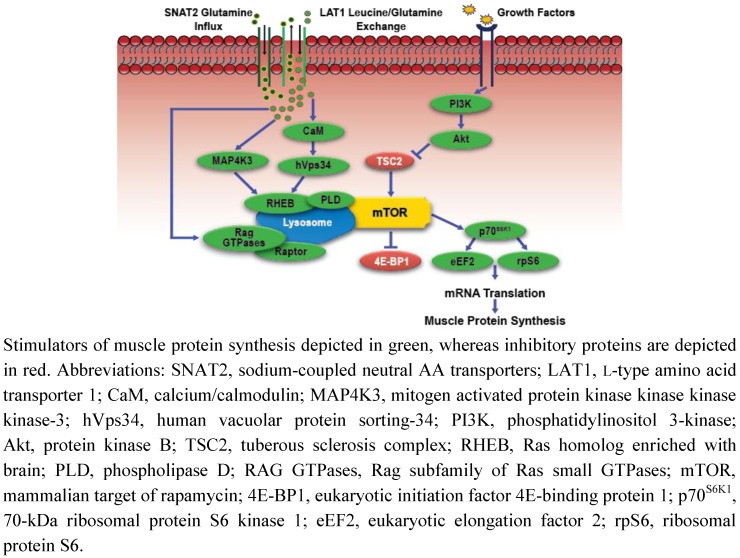
Simplified schematic of amino acid-mediated anabolic signaling.

Leucine is an established insulin secretagogue [68], and acute administration of excess leucine stimulates an increase in plasma insulin, resulting in enhanced mTORC1 signaling and MPS (Figure 2) [69,70]. The stimulation of MPS attributed to insulin occurs upstream of mTORC1 through PI3K and subsequent phosphorylation of protein kinase B (PKB/Akt). Under that regulatory control, Akt indirectly stimulates mTORC1 by phosphorylating TSC2, thereby activating the GTPase protein, Ras homolog enriched with brain (RHEB) [71]. Pharmacologic inhibition of insulin secretion fails to block leucine mediated mTORC1 signaling in laboratory animals [52,69]. These data suggest that AA stimulate anabolic signaling through both an insulin-dependent and independent mechanism. However, intracellular signaling and MPS responses to insulin inhibition were lower in comparison to animals with normal insulin function. As such, the degree to which leucine stimulates mTORC1 signaling may rely to an extent upon concomitant increases in insulin concentrations. More specifically, using an animal model, Crozier *et al*. [72] demonstrated that early stimulation of MPS in response to leucine administration was not reliant on insulin, but maximal stimulation of mTORC1 was dependent on elevations in circulating insulin concentrations. Pharmacologic-induced insulinemia in humans also fails to potentiate MPS responses to AA provision [73]. However, similar to the study by Crozier *et al*. [72], the stimulation of the mTORC1 was insulin-dependent, which suggest that a dissociative relationship between anabolic intracellular signaling and MPS responses to AA and insulin manipulations may exit [73]. 

*In vitro* studies suggest that hVps34 and MAP4K3 also contribute to insulin-independent, AA-induced regulation of mTORC1 signaling [6,7,9]. The hVps34 protein is a novel class 3 PIK that lies upstream of mTORC1. The activity of this lipid kinase is regulated in part by AA concentrations: hVps34 activity is attenuated with nutrient depletion, whereas increasing AA concentrations activates hVps34, phosphorylates p70^SK1^, and inhibits 4E-BP1 [6]. MacKenzie *et al*. [8] demonstrated that not only do contractile-induced elevations in leucine stimulate hVps34 activity, but sustained (3-h) provision of leucine to isolated muscle cells activates hVps34, which the authors suggest may prolong mTORC1 stimulation and contribute to long-term muscle hypertrophy. Gulati *et al*. [74] also demonstrated that elevations in AA concentrations modulate cellular calcium flux, causing the aforementioned CaM complex/Vps34 interaction and concomitant activation of mTORC1. In addition, MAP4K3, which was recently identified in *Drosophila*, is required for AA-induced stimulation of p70^SK1^ and 4E-BP1 through mTORC1 [9]. However, the mechanism by which MAP4K3 responds to fluctuations in AA concentrations to modulate mTORC1 activity is not well described. More importantly, not all studies support the interaction between these unique nutrient sensors, AA, and mTORC1 activity [75], and no studies have clearly identified the role of hVps34 and MAP4K3 in anabolic intracellular signaling and MPS in human skeletal muscle [76]. 

The functions of the Rag subfamily of Ras small GTPase proteins are associated with AA-mediated mTORC1 activation. Two independent groups of the four Rag GTPases that mammalian cells express (RagA, RagB, RagC, and RagD) modulate anabolic cell signaling through mTORC1 by functioning as heterodimers and binding with the mTORC1 associated protein Raptor. Raptor acts as an adaptor protein, recruiting substrates for mTORC1 phosphorylation, primarily p70^SK1^ and 4E-BP1 [10,66,77]. However, Rag interaction with mTORC1 does not directly activate the mTOR kinase; rather Rag proteins modulate cellular translocation of mTORC1 to the lysosome that facilitates an mTOR and RHEB interaction, which in the presence of elevated AA concentrations, results in RHEB-induced activation of mTORC1. Leucyl-tRNA synthetase (LRS) may be a novel AA sensing mechanism that functions in concert with Rag to activate mTORC1, as Han *et al*. [78] recently demonstrated that LRS binds directly with Rag, subsequently mediating GTPase activation and mTORC1 signaling in an AA-dependent manner. As such, Rag-mediated mTORC1 translocation and subsequent activation in response to AA sufficiency may prove to be the primary mechanism responsible for increased MPS following AA administration. 

Muscle intracellular AA transport proteins function as critical links connecting anabolic intracellular signaling to manipulations in AA concentrations by shuttling AA across cell membranes [79,80]. System L (L-type amino acid transporter 1(LAT1)/solute-linked carrier (SLC) 7A5 and CD98/SLC3A2 heterodimeric complex) and system A (sodium-coupled neutral AA transporters (SNAT2/SLC38A2)) transporters are involved in the regulation of intracellular AA concentrations by transporting large neutral AA (e.g., branched-chain amino acids) into muscle cells [81]. The expression and function of LAT1/CD98 and SNAT2 are positively associated with muscle growth, mTORC1 activation, and MPS [82,83]. In response to elevations in extracellular AA concentrations, SNAT2 increases glutamine influx, stimulating the LAT1/CD98-regulated bitransport system that exports glutamine out of the cell in exchange for leucine. 

Interactions between dietary AA manipulations and transporter-mediated anabolic intracellular signaling in human skeletal muscle have recently been investigated in a series of studies by Drummond *et al*. [11,12,67]. Intracellular leucine kinetics, MPS, mTORC1 signaling, and AA transporter expression were determined in healthy young adults before and after consuming a 10 g EAA supplement [12]. EAA ingestion resulted in increased leucine delivery to the muscle, intramuscular leucine concentrations, MPS, and mTORC1 activation. The anabolic response to EAA administration occurred with concomitant increases in LAT1 and SNAT2 expression suggesting a unique regulatory mechanism that may contribute AA-induced stimulation of MPS by enhancing the availability of AA. Drummond *et al*. [67] also demonstrated that 7 days of bed in older adults attenuates MPS, mTORC1 signaling, and LAT1 and SNAT2 protein content responses to EAA ingestion. These data demonstrate that relatively short periods of physical inactivity may contribute to age-related muscle loss by further desensitizing aging skeletal muscle to the potent anabolic stimulus of AA. Whether other conditions associated with muscle loss such as those experienced with weight loss in response to extended periods of energy deficiency result in similar patterns of AA transporter expression, mTORC1 signaling, and MPS responses to dietary AA manipulations has not been determined.

## 4. Integrated Regulation of Muscle Mass by Exercise and Amino Acids

**Figure 3:**
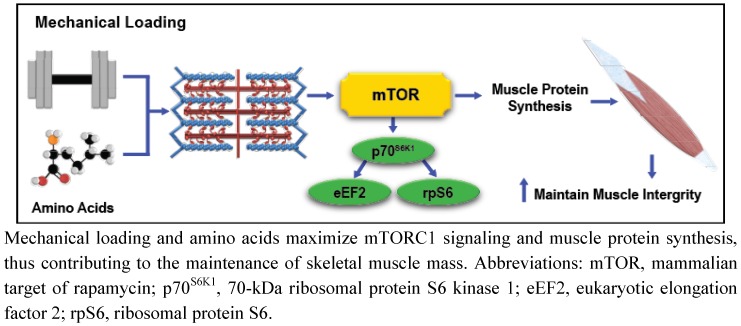
Simplified illustration of the combined effects of mechanotransduction and amino acid-mediated anabolic signaling on skeletal muscle integrity.

It is evident that exercise and AA stimulate mTORC1 independent of IGF-1/PI3K signaling through unique mechanotransduction and AA sensing mechanisms. However, mTORC1 signaling and subsequent MPS stimulation initiated by mechanical stress is more efficient in the presence of elevated AA concentrations [84]. As such, combining the metabolic effects of exercise with exogenous AA administration results in more a pronounced and sustained anabolic response than either stimulus can generally elicit alone, indicating that exercise and AA-mediated cell signaling are highly integrated. For example, mechanical loading through SAC-mediated calcium flux and enhanced intracellular AA concentrations both have the ability to activate the nutrient sensitive hVps34 kinase, stimulating RHEB-induced mTORC1 activation [6,7,8]. Furthermore, mechanical loading also modulates muscle intracellular membrane permeability to extracellular AA, which may enhance AA uptake [85], and stimulate mTORC1 signaling via hVps34, MAP4K3, and Rag GTPases-dependent mechanisms. Considering that exercise may independently modulate intracellular AA concentrations, the principal substrate for MPS, it is rather predictable that investigations consistently demonstrate enhanced mTORC1, MPS, and hypertrophic responses to mechanical stimuli when exercise is combined with AA supplementation (Figure 3) [1]. As such, exercise and nutrition strategies that effectively integrate mechanotransduction and AA-mediated intracellular signaling may conserve muscle health in populations predisposed to muscle loss. 

### Exercise, Amino Acids, and the Conservation of Muscle Mass

Reduced skeletal muscle mass coupled with an increase in body fat is among the most debilitating and consistent changes associated with aging. Age-related muscle loss is referred to as sarcopenia, which is clinically defined as presentation with muscle mass at least two standard deviations below the average muscle mass of a younger adult 35 years of age [86]. The rate of skeletal muscle loss can be much as 1% per year after 30 years of age, which continues until the end of life [87]. Although the etiology of sarcopenia is multifaceted, the most evident metabolic explanation for the decline in muscle mass is an imbalance between rates of MPS and proteolysis [88]. The reduction in muscle mass with aging can degrade strength, metabolic rate, aerobic capacity, and muscular function, thereby reducing quality of life [89], and predisposing elderly individuals to illness, fractures, and other chronic metabolic conditions such as obesity and type 2 diabetes [90], which ultimately increase mortality [91]. 

Multiple reports have demonstrated that aging skeletal muscle is desensitized to exogenous AA administration [61,92,93]. In fact, anabolic resistance to AA is considered a major contributor to age-related muscle loss [61]. However, this phenomenon may be overcome by manipulating the leucine content of an AA supplement or of a protein containing meal [61,62]. In a recent review, Breen and Phillips [94] hypothesized the existence of a leucine threshold, which must be surpassed to maximize MPS and mTORC1 signaling. They suggest that this threshold differs between young and aging muscle, where 1 g of leucine combined with resistance exercise in young adults is sufficient to stimulate MPS above resting conditions [56], but 2 g of leucine may be required to stimulate MPS above resting conditions in older adults after resistance exercise [95]. Interestingly, and in contrast to young adults [56], MPS in older adults reaches maximal stimulation after resistance exercise when approximately 4 g of leucine (40 g whey protein) is consumed. These findings are not only consistent with earlier reports in older adults [61,62] but also similar to recent data from our laboratory that suggest that leucine requirements may not only be dependent on age but also perhaps by the metabolic demand for energy and AA [60]. 

Evidence also demonstrates that MPS and mTORC1 signaling responses to resistance exercise are blunted in older compared to younger adults [96,97,98]. A recent report suggests that AA transporter expression may be a contributing factor to age-related anabolic resistance to mechanical loading [11]. Drummond *et al*. [11] demonstrated increased LAT1 expression with concomitant elevations in mTORC1 signaling in young adults, yet no changes in transporter expression and anabolic signaling in older adults following a bout of resistance exercise. However, manipulating the volume of mechanical overload may prove to be an effective compensatory strategy against anabolic resistance to exercise, which may contribute to the conservation of muscle mass [96,97]. Fry *et al*. [99] demonstrated that combining low-intensity, high-volume resistance exercise with blood flow restriction (*i.e.*, leg blood flow was restricted using a lower extremity pressure cuff; Kaatsu-Master Mini, Tokyo, Japan), stimulated marked increases in MPS and mTORC1 signaling in older adults; although the practical applications (e.g., access to pressure cuffs and potential for discomfort) of blood flow restricted exercise may be limiting. Other studies recommend that older adults perform low-intensity, high-volume resistance exercise to muscle failure to impose a cumulative mechanical load that is greater than those typically observed with high-intensity, low-volume resistance exercise [18]. Therefore, low-intensity, high-volume exercise to muscle failure or perhaps when safely combined with blood flow restriction may be an effective anabolic stimulus and form of exercise therapy for older adults to perform in comparison to conventional resistance exercise training [97]. Furthermore, the most effective strategy against age-related muscle loss is arguably the combination of exercise with AA (or high-quality protein) supplementation, as there is no shortage of reports that demonstrate acute mTORC1 and MPS responses to exercise and AA and protein consumption are more pronounced in older adults when these anabolic stimuli are combined [11,12,76,95,100]. Whether acute anabolic intracellular signaling and MPS responses result in long-term conservation of muscle mass following structured, low-intensity, high-volume exercise training with optimized nutritional support has not been determined. 

## 5. Conclusions

The distinct metabolic effects by which exercise and AA modulate anabolic intracellular signaling, MPS, and contribute to the regulation of muscle mass have been studied extensively in recent years. Intracellular calcium concentrations mediated by SAC and PA accumulation triggered by PLD have been identified as distinct mechanosensors stimulating mTORC1 activity in response to mechanical stress. Nutrient sensing mechanisms including hVps34, MAP4K3, and the Rag GTPases appear to modulate mTORC1 localization and activation in response to elevations in AA concentrations. The expression and activity of multiple intracellular AA transport proteins may also serve a critical role in regulating anabolic responses to AA supplementation by enhancing the availability of leucine. Interestingly, exercise also shares the ability to alter intracellular AA concentrations and subsequent mTORC1 signaling through similar nutrient sensitive mechanisms. Certainly, further study is necessary to confirm the function of these novel mechanotransduction and AA-sensing mechanisms demonstrated in *in vitro* and *in vivo* animal studies on human muscle protein metabolism in response to a variety of physiological stressors. Regardless of the mechanism, it is clear that exercise and exogenous AA supplementation are potent anabolic stimuli that can be used successfully as integrated therapies to promote muscle health in populations susceptible to muscle loss. 
